# Using climax thinking to understand psychological drivers of attitudes towards two coastal climate adaptations around the hypertidal Bay of Fundy

**DOI:** 10.1007/s44218-026-00163-8

**Published:** 2026-10-30

**Authors:** Samantha Howard, Brooke McWherter, K. L. Akerlof, Kate Sherren

**Affiliations:** 1 School for Resource and Environmental Studies, Dalhousie University, Halifax, NS Canada; 2 Department of Environmental Science and Policy, George Mason University, Fairfax, VA USA

**Keywords:** Managed dike realignment, Flood risk mapping, Nature-based solutions, Ordered logit model, Quantitative survey, Environmental psychology

## Abstract

Coastal climate adaptations are changes that tackle the challenges arising from sea level rise. Adaptations such as risk information and changes to public infrastructure have been met with some degree of public opposition. Climax thinking is an emerging framework for exploring this resistance. In this study, we used climax thinking to explore public responses to two specific coastal climate adaptation strategies: public flood risk mapping and managed dyke realignment. Using a survey administered to residents around the hypertidal Bay of Fundy, in Atlantic Canada, we found low knowledge of both. While most residents supported each, 30% expressed resistance to flood risk mapping based on real estate value concerns, and 20% expressed resistance to managed dyke realignment that might involve retreating homes or businesses. Using ordered logit models we tested the dimensions of this resistance (deploying Time Perception, Futures Consciousness, Basic Values, and Awareness of Consequences) as well as risk perceptions. Only future orientation was significant (reducing resistance) for both models, supporting its power for climate action. Other significant variables differed by the type of adaptation, showing the importance of asking about adaptation options separately, rather than climate action in general. For instance, a past focus associated with a sense of loss was shown to increase resistance for managed dyke realignment. The research suggests new areas of exploration for climax thinking, including the scale and circumstances in which it manifests (e.g., proximity, impact, novelty), and the value of exploring relational dimensions.

**Supplementary Information:**

The online version contains supplementary material available at https://doi.org/10.1007/s44218-026-00163-8.

## Introduction

Coastal climate change adaptations are changes to socio-cultural contexts to reduce exposure and vulnerability to the challenges arising from climate change impacts to coastal environments, including sea level rise, flooding and erosion (Adger et al. 2005). Such adaptations may be incremental, reinforcing the existing system, or transformative, altering its structure and function, and can take economic, physical, technical or mindset (cognitive-psychosocial) forms (Glavovic et al. 2022; Wamsler 2018). Nature-based coastal adaptations are generally described in terms of physical interventions that leverage natural materials to protect coastal residents, infrastructure and ecosystems (van der Meulen et al. 2022). Recently, Sherren et al. (2024) argued to include cognitive shifts within the nature-based adaptation concept, helping people make head space (as well as physical space) for coastal dynamics. Such collective reimagination could potentially support the acceptance (and adoption) of publicly available flood risk mapping (Henstra et al. 2019), managed retreat, and restoration of tidal wetlands for nature-based coastal protection (International Union for Conservation of Nature 2020; Vouk et al. 2021).

Resistance to coastal climate adaptations is a well-documented phenomenon, yet the reasons remain inadequately understood. van Valkengoed and Steg (2019) performed a meta-analysis to explore individual motivations for personal adaptation behaviours such as preparatory action, purchasing insurance, seeking information, evacuating and supporting adaptive policies. Of the 13 tested, the strongest factors were: feeling capable of doing the action (self-efficacy), feeling badly for not doing the action (negative affect), believing others are doing the action (descriptive norms), and believing the action will protect them (outcome efficacy). More recently, Bax et al. (2025) found local support for specific managed dyke realignment (MDR) sites to be best explained by trust in the institutions running the projects, expectations of positive outcomes from the project, and conducive emotions. Sarabi et al. (2020) found that public resistance to the implementation of nature-based solutions such as dyke realignment emerges, for instance, due to a lack of public awareness of adaptation strategies, or generalized resistance to change. An emerging idea in understanding generalized resistance to change is climax thinking. Climax thinking borrows ideas from succession ecology to describe a tendency for individuals to perceive the current landscape as being in its final and ideal form, leading to difficulty conceptualizing alternative options (Sherren 2021). In this study we set out to understand resistance to coastal climate adaptation by integrating established psychological theories and tested question sets that align with climax thinking dimensions, using a mail-out multi-reminder survey around the Bay of Fundy, Atlantic Canada. We explored attitudes towards managed dyke realignment (MDR) and publicly available flood risk mapping (FRM).

This study undertakes theory-building to advance understanding of climax thinking by integrating established psychological factors to understand resistance to two climate adaptations, but we have equal interest in how the people of this region perceive these options. This study was guided by the following questions:

1. How do residents perceive the coastal climate adaptation strategies of managed dyke realignment and publicly available flood risk mapping?
2. How do psychological concepts related to climax thinking influence resistance to those coastal climate adaptations?

## Background

### Understanding climax thinking as a driver of resistance to nature-based adaptation

Climax thinking has been conceptualized as driven by both spatial and temporal dimensions and associated causes including exceptionalism and uncertainty (Sherren 2021). The spatial axis represents the perception of change as affecting the individual (i.e., close to self) or as a distant concern (i.e., other). On that axis from self to other, climax thinkers would be oriented toward the self. As a result, climax thinkers are more concerned with their own landscapes in their current state, and they might not recognize or care that rejecting local change could have consequences for those further away from themselves. For example, they may not be concerned that putting up sea walls on their land may worsen erosion for their neighbors, or that rejecting wind turbines in their community could perpetuate coal production elsewhere. The temporal axis of climax thinking considers how individuals perceive change to impact existing conceptualizations of their landscapes. Climax thinkers have been assumed to be present-oriented, concerned with maintaining stability and sustaining their current landscapes. However, there may also be complex past-orientations depending on whether present landscapes are entangled with how their ancestors have used them, consistent with Eaton et al. (2021) concept of cultural counterpoints. As a result, climax thinkers might reject significant landscape changes even if the changes are intended to avoid worse impacts in the future. For example, they may reject efforts to protect homes from flooding or to retreat to less flood-prone areas even if inaction is likely to result in extensive repairs or personal risks later should flooding occur (Sherren 2021).

Novel climax thinking measures have previously been piloted using surveys to explore resistance to land-use changes such as wind farms and nature-based coastal adaptation (Chappell et al. 2020; Sherren et al. 2022). Those studies suggest climax thinking is a construct not easily measured directly, meaning we can only measure it by knowing the ‘symptoms’ of a climax thinker, such as underlying spatial and temporal psychological drivers (Sherren et al. 2022). In that pilot work, statements about self-orientation repeatedly demonstrated the best predictive power for resistance to climate adaptation. For example, including the statements, “I am not able to cope with the land changes required to deal with significant increases in flood risk at this point in my life” and “Flood management decisions I make do not have implications for others” almost quadrupled the predictive power of a model to explain resistance to public FRM in a different region (Howard and Sherren 2023). Similarly, experimental focus groups employing priming techniques to see what influenced support for nature-based coastal adaptation showed that future- and other-oriented priming devices *reduced* post-test measures of climax thinking and increased overall proactivity in discussions of climate change (Sherren et al. 2022).

Climax thinking aligns with well-established psychosocial theories that describe how temporal and spatial orientations inform responses to management strategies. Prior research indicates that resistance to landscape transformation is influenced by individuals’ underlying time orientations, value priorities and beliefs about consequences for others or the natural environment. Theories such as Time Perspective and Futures Consciousness explain temporal factors, how people emphasize the past, present, or future and whether they perceive agency and interdependence in environmental systems (Ahvenharju et al. 2018; Lalot et al. 2020). Other frameworks such as Value-Belief-Norm theory and Basic Human Values are useful in understanding elements of both spatial and social orientations, including whether individuals prioritize self-interest, others, or biospheric concerns (Gärling et al. 2003; Hansla et al. 2008; Schwartz 2012; Stern et al. 1999).

Risk perception theories offer an additional potential predictor for operationalizing climax thinking (Hügel and Davies 2020; Keller et al. 2012; Wachinger et al. 2013). Risk perception is commonly understood as an individual’s assessment of the perceived probability and severity of a hazard and has been used to explore acceptance of risk in the face of landscape changes and natural disasters. This can help us understand how future environmental conditions are perceived, and whether a presence or a lack of risk perception may contribute to an individual’s motivation to adapt (Hügel and Davies 2020; Wachinger et al. 2013). Across a variety of environmental risks, including floods, researchers have found that risk perception is heavily influenced by both personal experience with a previous environmental hazard, and level of trust in decision making authorities who act to mediate environmental risks. There is a notable paradox within environmental risk perception literature, where higher risk perception does not reliably lead to increased preparedness for or adaptiveness to future environmental disasters (Wachinger et al. 2013). These theoretical perspectives offer conceptual mechanisms that can help explain variation in climax thinking and identify drivers of resistance to climate adaptation strategies.

### Nature-based adaptation around the Bay of Fundy

**Fig. 1:**
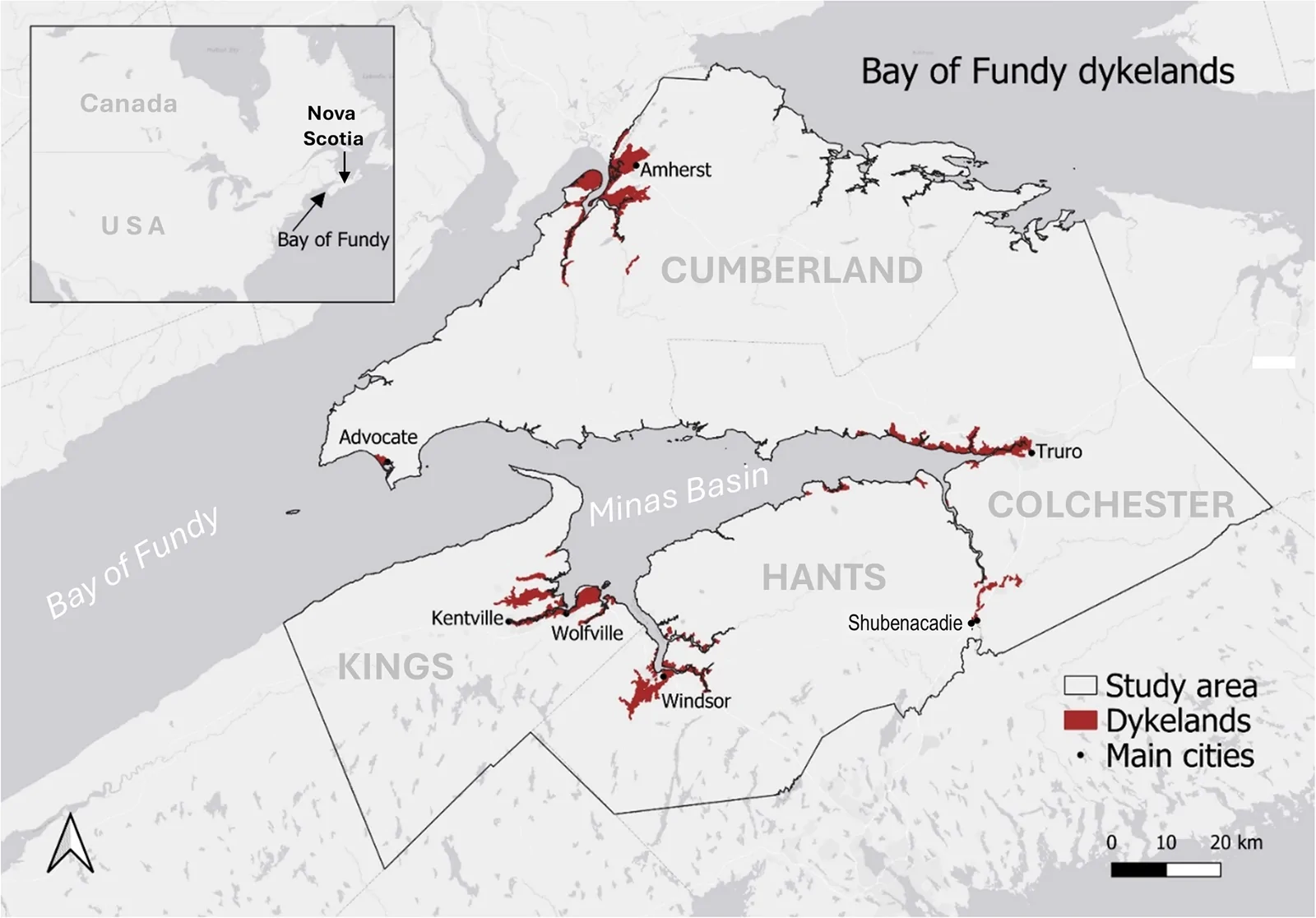
Study Area Overview

The Bay of Fundy—located between Nova Scotia and New Brunswick on the east coast of Canada at the top of the Gulf of Maine—represents a key region where changing climatic conditions are driving interest in mitigation and adaptation management and policy strategies (Fig. 1). The Bay of Fundy region is known for the largest tides in the world, with the average tidal range being 13 m (NOAA 2023). At the head of the Bay of Fundy, the Minas Basin experiences particularly dramatic tidal ranges with water levels fluctuating up to 16 m every 12 h (NOAA 2023). Two nature-based adaptation strategies of relevance to this region are managed dyke realignment and flood risk mapping.

#### Managed dyke realignment

Early French settlers (called Acadians) to the Bay of Fundy region in the 1600s created dykelands (called dikelands or polders elsewhere) by enclosing tidal wetlands using earthen dykes and draining rainwater through ditches and one-way gates to create fertile farmland (Bleakney 2004; Rudin 2021). While useful for creating farmland in a place not otherwise blessed with good soil, dykeland development led to lost ecosystem services due to the removal of around 69% of the Bay of Fundy’s coastal tidal wetlands (Sherren et al. 2021b). Today approximately 70% of dykelands continue to be actively farmed in Nova Scotia even as they are threatened by climate change. In Nova Scotia, as around the world, dykes are at risk of overtopping and seepage due to sea level rise and storm action. Dykes require intervention to keep the lands they protect above water (Bax et al. 2023), though dykelands with intact dykes can still be affected by overland flooding during high tide and saltwater intrusion. In many regions, maintaining these extensive historical structures represents technical, logistical, and economic challenges. For instance, maintaining the 240 km of dykes on the Nova Scotia side of the Bay of Fundy is both technically and economically unrealistic, due in part to coastal squeeze on the foreshore, meaning there is not enough space available to widen dykes enough to allow them to be built higher in situ (van Proosdij et al. 2018).

Managed dyke realignment (MDR) is a nature-based adaptation that involves retreating hard infrastructure landward, for instance building higher dykes further inland and facilitating areas left on the water side of the new dykes to return to tidal wetlands, or converting them to managed living shorelines (Estevez and Williams 2017). In Canada, there has been resistance to retreat-based strategies such as dyke realignment, due to significant cultural attachment to both the coastal areas and landscapes the dykelands create, and the dyke infrastructure itself (Mallette et al. 2021).

#### Public flood-risk mapping

As climate change impacts accelerate globally, flood events are predicted to increase in frequency and severity, and will require adaptation and mitigation measures to manage effectively (IPCC 2023). Flooding events across Canadian coasts have been increasing in frequency and severity, resulting in devastating losses of lives, homes, and infrastructure (MacDonald and Smellie 2021; Schmunk 2021). While flooding is a natural process, human activities that worsen climate change and increase exposure through development are accelerating the occurrence and increasing the severity of flooding events across the planet (Hirabayashi et al. 2013). In 2019, the Canadian government declared a climate emergency in recognition of the need for climate mitigation and adaptation (Jackson 2019).

As in many coastal regions, the Bay of Fundy Region has extreme flood risk. The watershed area of the Minas Basin covers about 20% of mainland Nova Scotia and is roughly 5 times larger in surface area than the basin itself, making the region particularly susceptible both to flooding from the outer Bay of Fundy and overland from the watershed. The region is projected to experience some of the greatest relative sea level rise of all Canadian coastlines, with an expected average increase of 77–120 cm by the year 2100 (James et al. 2015). This higher relative sea level rise in this region is caused by sea level rise combined with glacial-isostatic adjustment, which is causing the land to subside (Bush et al. 2019).

The Minas Basin region experiences significant annual flooding, most of which is coastal or pluvial in nature. Coastal flooding, primarily from storm surges and hurricanes, has damaged infrastructure across the region in recent events, including Hurricane Fiona in 2022 and Hurricane Dorian in 2019. Pluvial flooding, which is a result of rainfall or snowmelt, occurs annually during the springtime throughout the Bay of Fundy watershed region, and occasionally at other times of the year, washing out roads, damaging infrastructure, and isolating people in their homes (Starratt 2022). In 2023 and 2024, fatal flooding events occurred in the region (after this research was conducted). Flooding is particularly challenging in dykeland areas if the rainfall occurs at high tide, when the dyke gates are closed, trapping rainwater behind dykes.

Yet the Canadian media contains numerous references to public alarm following the release of FRM to the public. Many public mapping resources have been released only to be quickly withdrawn after public protest. Previous research in Nova Scotia shows that some respondents believe publicly available FRM presents too big a risk for landowners’ property values (Howard and Sherren 2023). Canadians living in high flood-risk areas have expressed a lack of willingness to pay personally for flood protections and recovery, putting pressure on the public purse (Thistlethwaite et al. 2018). Increasing accessibility and availability of flood maps is essential for private and public benefits such as better emergency preparedness, and equitable housing transactions (Lyle et al. 2024).

## Methods

### Study area

Our study area focused on the counties of Hants, Kings, Cumberland, and Colchester that surround the Minas Basin (Fig. 1). These counties include the largest towns on the Nova Scotian side of the Bay of Fundy coast, for instance Amherst (population 10,000), Truro (13,500), and Kentville/Wolfville (13,000), but also more rural inland areas. Multiple mailout surveys were deployed containing established psychological constructs aligned with the drivers of climax thinking and were distributed to a probabilistic sample of households in the counties listed above. Deploying a survey in this area provided an opportunity to get a population-level understanding of the issues, including people who had varied experiences with floods and dykelands, living in low-lying or higher areas, all of whom may be impacted by coastal and dykeland management decisions.

### Survey design

This was an omnibus survey serving multiple purposes, but this study focuses on a subset of 45 questions. Attitudes towards FRM and MDR were selected as proxies for resistance to change (i.e., climax thinking). One measure was retained for analysis for each management strategy based on the highest response rate and its statistical suitability (i.e., non-skewed distribution). Explanations and images of FRM and MDR were provided in advance of any questions about each approach (Supplemental Box 1), and respondents were also immediately asked after the appropriate explanation whether they had previously seen a flood risk map for their region or heard of MDR.

Theoretical variables to operationalize the dimensions of climax thinking that were found useful in other work were taken from pre-validated scales (Table 1). This included: two statements from Awareness of Consequences to measure self-orientation and one to measure nature-orientation (Gärling et al. 2003); two statements from the Schwartz Portrait Values Questionnaire to measure other-orientation and one to measure nature-orientation (Schwartz 1994); three statements from the Futures Consciousness scale to measure future-orientation (Lalot et al. 2020); and two statements from the Zimbardo Time Perspective Inventory to measure past-orientation (Zimbardo and Boyd 1999). Flood risk perception was measured with four statements each about perceived likelihood and severity of flooding impacts (Harlan et al. 2019) (see Table 2). For climate change concern we used standard statements from the Climate Change in the American Mind nationally representative surveys conducted by the Yale Program on Climate Change Communication and the George Mason University Center for Climate Change Communication (Myers et al. 2013).

**Table 1:** Operationalization of climax thinking and risk perception dimensions, as well as control variables, in MDR and FRM models

Concept	Source	Short form	Statement (scale)	MDR	FRM
**Psychology**					
Self-orientation	Awareness of consequences (Gärling et al. 2003)	Threaten jobs	Protecting the environment will threaten jobs for people like me. *(5-point Agree Likert)*	X	X
		Limit my choices	Laws to protect the environment will limit my choices and personal freedom. *(5-point Agree Likert)*		X
Other-orientation	Schwartz Basic Values (Schwartz 1994) universalism and benevolence items from Portrait Values Questionnaire (“How much is this person like me?”)	Everyone treated equally	They think it is important that every person in the world be treated equally. They believe everyone should have equal opportunities in life. *(Portrait values options: Not like me at all/ A little like me/ Somewhat like me/ Like me/ Very much like me)*	X	X
		Care for others	It’s very important to them to help the people around them. They want to care for the well-being of others. *(Portrait values)*		X
Future-orientation	Futures consciousness (Lalot et al. 2020). (Cronbach’s alpha = 0.68)	Futures consciousness	I think about how things might be in the future I am willing to sacrifice my immediate happiness or well-being in order to achieve something in the future. I consider how things might be in the future, and try to influence those things with my day to day behavior *(all 5-point Agree Likert)*	X	X
Past-orientation	Past-negative and past-positive statements from the Zimbardo Time Perspective Inventory (Zimbardo and Boyd 1999)	Missed out on good things (reversed)	I think about the good things I have missed out on in my life *(5-point Agree Likert)*	X	X
		Childhood good memories	Familiar childhood sights, sounds, smells often bring back a flood of wonderful memories *(5-point Agree Likert)*		
Nature-orientation	Schwartz Basic Values Portrait Values Questionnaire item for Universalism	Care for nature	They strongly believe that people should care for nature. Looking after the environment is important to them. *(Portrait values)*	X	X
	Awareness of Consequences	Species become extinct	The next several decades, thousands of species will become extinct. *(5-point Agree Likert)*	X	X
**Awareness**					
Climate concern	Standard questions from Climate Change in the American Mind, an annual survey conducted jointly by the Yale Program on Climate Change Communication and the George Mason University Center for Climate Change Communication (Myers et al. 2013) Climate change variables were turned into Z-scores and averaged due to different scales (Cronbach’s alpha = 0.81)	Climate change concern	How important is the issue of climate change to you personally? *(5-point scale from Extremely important to extremely unimportant)* How worried are you about climate change? *(4-point scale from Very worried to Not worried at all)* How much do you think climate change will harm you personally? *(4-point scale from A great deal to Not at all)* How much do you think climate change will harm future generations? *(same as above)*	X	X
	Awareness of Consequences	Defer responsibility to future	We don’t need to worry much about the environment because future generations will be better able to deal with these problems than we are. *(5-point Agree Likert)*	X	X
Flood risk likelihood	Likelihood of flooding impacts from the Perceived risk of future flooding events scale (Harlan et al. 2019)	Flooding damage home	Suppose a flood occurred in your region within the next five years. How likely do you think it would be that your home would be seriously damaged or destroyed*? (4-point from Not likely at all*,* Unlikely*,* Somewhat likely*,* Extremely likely)*		X
		Flooding hurt loved ones	Suppose a flood occurred in your region within the next five years. How likely do think you it would be that you or some of your loved ones would be hurt? *(same as above)*		X
Familiarity with adaptations		Seen flood map	Have you ever seen a flood risk map for your region? *(yes/no)*		X
		Heard of MDR	Have you heard of managed realignment before? *(yes/no)*	X	
**Controls**					
Gender		Is a male	Gender *(female*,* male*,* neither/third gender*,* prefer not to say)*	X	X
Education		Education	Highest level of education *(numerous options grouped into High school or lower*,* Some college*,* Some university)*	X	X
Economic alignment		Free-market preferences	Where would you place yourself on a scale from 1–10, with 1 being the desire for the economy to be run by a cooperative collective agency (i.e., government), and 10 being the desire for the economy to be left to its own devices (i.e., market).	X	X
Grandchildren		Has grandchildren	Do you have any grandchildren?	X	X

**Table 2:** Perceptions of managed dyke realignment and flood risk mapping. Participants were asked to rate their level of agreement with each statement on a 5-point Likert Scale, which was then aggregated to the three levels below, with Strongly Agree and Agree being combined to Agree, and Strongly Disagree and Disagree combined to Disagree

Adaptation	Statement	Agree	Neutral	Disagree
Managed dyke realignment (*N* = 219)	Dykes in Nova Scotia should be raised to counter storm surges and rising sea levels.	73%	22%	5%
	Restoring tidal wetlands would help protect Nova Scotia against storm surges and sea-level rise.	74%	24%	3%
	Moving dykes back will allow us to keep protecting useful dykeland while providing space for flood water.	69%	26%	5%
	Restoring dykelands to tidal wetlands represents more risk than benefits.	10%	57%	33%
	In some cases, it would be acceptable to relocate homes or businesses to allow for dyke realignment.*	36%	44%	20%
Flood risk mapping (*N* = 212)	Flood risk mapping for a given property should only be available to the specific landowner(s).	31%	12%	57%
	Publicly available flood risk mapping would have an unacceptable impact on the real estate value of affected landowners.*	30%	31%	39%
	Flood risk mapping for properties in my region should be publicly available for all to use.	85%	10%	5%
	Property-level flood risk mapping provides important information for current and future home buyers and renters.	93%	5%	2%

*Denotes those used as response variables in the regression

Additionally, while demographic variables were collected on age, gender, highest level of education, and average annual household income, only gender and education were retained due to low response rates among the other variables. Two additional control variables were included in the analysis. We asked people if they had grandchildren to expand on prior work that tested having children as a predictor on resistance to FRM (Howard and Sherren 2023). We also asked about preferences for free markets, specifically whether they would like the economy to be run by the government or the market, because much climate change response calls for public investment, whether for mitigation, adaptation or recovery.

### Survey implementation

The survey was pre-tested with several people who live in the region and some revisions were made. Simple random sampling was used to obtain a sample of single-family dwelling residential addresses located within the Minas Basin study area (Fig. 1). This sample was selected from addresses on the Residential Dwellings Characteristics dataset on January 10th, 2022 (datazONE 2022). As this dataset does not contain postal code information, the NS postal code dataset was obtained from the Dalhousie University GIS Centre, using version 2019.3 from DMTI Spatial. The focus on detached house addresses created additional bias in the final sample by excluding apartment or institutional residents from responding, but at the time it was the only way to create a probabilistic sample of this rural area for mailout and tracking responses.

The survey was distributed using a hybrid multiple mail-out survey method, adapted from Dillman (1978). A total of three mailouts were sent over the course of a four-month recruitment period. The first mailout was a letter sent on June 6, 2022, the second mailout was a postcard sent on July 8, 2022, and a final letter was sent on September 2, 2022. The survey was hosted online, using Qualtrics software. All three survey invitations received by participants included both a QR code and a legible link to access the survey. Addresses were only sent a reminder if they did not complete the survey, although slow turnaround at Canada Post in relation to undeliverable mail was a significant challenge. Completion was identified by requiring participants to enter a unique code printed on their mailed-out invitation.

The contents and collection of the survey were approved by the Dalhousie University Research Ethics Board prior to distribution (REB# 2022–6088). The first 100 participants who responded to the survey had the option to enter a drawing for 1 of 10 $50 Irving Gas or Tim Hortons coffeeshop gift cards, and all remaining participants had the option to enter a draw for 1 of 10 $25 Irving Gas or Tim Hortons gift cards (choice of retailer was left to the participant).

### Data analysis

Diagnostic tests and visual inspection of the residuals suggested that an ordered logit (ologit) model with robust standard errors to account for heteroskedasticity was the best fit. We estimated two ologit models to examine predictors of adaptation-related attitude. For each model, the specifications were as follows with FRisk included only in Model 2 (FRM):

$$\begin{aligned}\mathrm{log}\left(\frac{P\left(RM\right)\geq{j}}{P\left(RM\right)<j}\right)=&\;\alpha_{j}-\left(\beta_{1}\mathrm{Self}+\beta_{2}\mathrm{Other}+\beta_{3}\mathrm{Future}\right.\\&\left.+\beta_{4}\mathrm{Past}+\beta_{5}\mathrm{Nature}+\beta_{6}\mathrm{Fam}\right.\\&\left.+\beta_{7}\mathrm{Concern}+\beta_{8}\mathrm{FRisk}+\beta_{9}\mathrm{Demo}\right),\\&j=1,\cdots{\cdot},J-1\end{aligned}$$

The dependent variables (RM) represent agreement with one of two management-impact statements, using the five-point scale (collapsed to three for presenting descriptive results).

- Model 1 (MDR) uses “In some cases, it would be acceptable to relocate homes or businesses to allow for dyke realignment” (1 = strongly agree). This statement was chosen (but reversed to reflect resistance) as it elicited the greatest diversity of opinions among the five tested, with responses spread across all categories rather than heavily skewed (Table 2; Supplemental Fig. 1a).
- Model 2 (FRM) uses “Publicly available flood risk mapping would have an unacceptable impact on the real estate value of affected landowners” (5 = strongly agree). This statement also sparked the widest range of opinions from the four options given and was also related to homes (Table 2; Supplemental Fig. 1b).

More variables were required to build a strong model for FRM than for MDR (Table 3). Self-orientation (SELF) includes one item in MDR (“protecting the environment will threaten jobs for people like me”) and two items in FRM, with the added agreement that “Laws to protect the environment will limit my choices and personal freedom” (5 = strongly agree). Other-orientation (OTHER) includes one shared item (“every person in the world should be treated equitably”) and, in FRM only, an additional item reflecting the belief that “it is important to help others around me”. Future-orientation (FUTURE) is an index calculated as the average of the three Likert items reflecting future-focused thinking and willingness to sacrifice now for future outcomes (Cronbach’s alpha = 0.68). Past-orientation (PAST) is a single item measuring disagreement with dwelling on “good things missed out on in life” for MDR, but a second item in FRM is about “good childhood memories”. Nature-orientation (NATURE) is a two-item vector capturing the belief that people should care for nature and that species are going extinct.

**Table 3:** Ordered logit models for MDR and FRM, showing log-odds co-efficients. Robust standard errors in parentheses. Full statements and response options in Table 2

Concept	Variables	MDR	FRM
Self-orientation	Threaten jobs	0.0383 (0.197)	0.397* (0.22)
	Limit my choices	-	-0.105 (0.171)
Other-orientation	Everyone treated equally	0.281** (0.133)	-0.207 (0.163)
	Care for others		-0.142 (0.135)
Future-orientation	Futures consciousness	-0.796** (0.344)	-0.753** (0.344)
Past orientation	Missed out on good things	0.382* (0.213)	0.198 (0.185)
	Childhood good memories	-	-0.121 (0.195)
Nature-orientation	Care for nature	0.0341 (0.154)	-0.0389 (0.196)
	Species become extinct	-0.708*** (0.204)	0.301 (0.203)
Climate concern	Climate change concern	-0.0666 (0.294)	0.241 (0.241)
	Defer responsibility to future	-0.19 (0.234)	-0.118 (0.194)
Awareness	Heard of MDR	-0.654* (0.372)	-
	Seen flood map		0.148 (0.428)
Flood risk likelihood	Flooding damage home		0.641** (0.253)
	Flooding hurt loved ones		-0.451 (0.291)
Control	Is a male	-0.831** (0.344)	0.00521 (0.381)
	Free-market preferences	0.044 (0.073)	-0.0416 (0.0833)
	Has grandchildren	0.211 (0.309)	0.798** (0.366)
	*Compared with Education = 1*,* High school or lower*:		
	Education = 2, Some university	-0.138 (0.391)	-0.121 (0.489)
	Education = 3, Some college	-0.342 (0.433)	0.147 (0.524)
	/cut1	-7.074*** (1.822)	-4.863*** (1.69)
	/cut2	-4.689*** (1.809)	-3.169* (1.683)
	/cut3	-2.458** (1.777)	-1.533 (1.660)
	/cut4	-0.837 (1.764)	0.853 (1.584)
	Observations	178	180
	Wald chi2(14)	38.23	42.41
	Prob > chi2 (p-value)	0.0005	0.0016
	McFadden Pseudo R^2^	0.0864	0.0823
	McKelvey & Zavoinas R^2^	0.22	0.225
	Log pseudolikelihood	-218.07	-241.823

*** *p* < 0.01, ** *p* < 0.05, * *p* < 0.1

Non-climax variables were also included. Familiarity (FAM) represents one of two binary variables of having heard of dyke realignment (MDR) or having seen a flood risk map for one’s region (FRM). Climate concern (CONCERN) is an index created by converting four climate-related variables to Z-scores and averaging them (personal importance of climate change, worry about climate change, belief that climate change will harm the respondent personally, and expected harm to future generations; Cronbach’s alpha = 0.86). FRM contains an additional two-item vector measuring perceived flood risk to home and to loved ones (FRISK). Both models include a demographic vector (DEMO) with indicators for gender (1 = man), having grandchildren (1 = yes), education (dummy variables for university and college, compared with high school or less) and free-market preferences ranging from 1 (“run by the government) to 10 (“market”).

Diagnostics for the final ologit models focused on overall model fit. Likelihood ratio chi-square tests showed that each model significantly improved relative to intercept-only specification. The Brant test could not be computed due to sparse cells underlying the binary logits, but the model converged normally with stable parameter estimates. Given the ordered structure of the outcome variables and the acceptable fit of the full specifications, the ologit framework was retained.

## Results

### Overview

The survey was sent to a random sample of 1350 addresses and received 233 responses with 217 of those being complete enough to include in the ologit models, using a cut-off of no more than 12 unanswered questions. Due to our sampling frame, 233 addresses were also returned as undeliverable, leading to a response rate of 21% at a margin of error of ± 6% at a 95% confidence level. Listwise deletion was used for missing data, leading to model observations of 178 (MDR) and 180 (FRM) as reported in the results, instead of 217.

Representativeness was determined by comparing demographic factors of gender, income, and education to the 2021 Canadian census for the counties of Hants, Kings, Cumberland, and Colchester. Within our sample, there was an overrepresentation of those with a higher income than the regional average, and an older average age (54) than the regional average. Many of our respondents (49%) were between the ages of 50 and 70, which represent only 38% of the population. Our sample was skewed (66%) female and generally had more post-secondary education than the average population of the area. Our sample was over-representative of those earning a higher income, with 32% of those who responded earning over $100,000 in household income, while that only represents 5% of the population (Supplemental Table 1).

We do not use weighting to remove these differences but discuss the implications of this lack of representation in the discussion section. Robustness checks suggest that age, income and education do not statistically differ across resistance levels for either MDR or FRM. A descriptive comparison suggested possible gender differences for MDR resistance (Supplemental Table 2a), and regression analyses indicated the effect was statistically significant (*p* = 0.016). Similarly, age showed a marginal relationship with FRM (Supplemental Table 2b); however, age was not retained in our final regression models.

### Perceptions of climate change, risks and adaptations

Climate change concern was extremely high overall within the sample (Supplemental Table 3). Almost three-quarters (72%) of respondents said that climate change was extremely or very important to them personally, with only 7% saying it was not important. 90% were very or somewhat worried about climate change. Most respondents felt that climate change would personally harm them only a moderate amount (58%), with a further 18% believing there would be a great deal of harm. But there was wide agreement (80%) that climate change would harm future generations a great deal, and wide disagreement (89%) that they didn’t “need to worry much about the environment because future generations will be better able to deal with these problems”. Flood risk perceptions were slightly lower but still felt by the majority: 61% of respondents felt that if a flood was to occur in their region within the next five years that it was extremely or somewhat likely that their home would be seriously damaged or destroyed, and 56% that they or their loved ones would be hurt (Supplemental Table 3).

Familiarity with MDR and publicly available FRM were both low: only 20% of respondents had seen a flood risk map for their area and 23% had ever heard of MDR. Respondents exhibited agreement to many of the statements for both FRM and MDR, even when they contradicted each other (Table 2). For instance, there was strong agreement that dykes should be raised to counter storm surges and rising sea levels, *and* that restoring tidal wetlands would help protect against storm surges and sea-level rise. The greatest diversity of responses for both climate adaptations was present when asking about impacts on homes or real estate: 20% disagreed that it may be acceptable to move homes or businesses to allow for MDR, and 30% agreed that publicly available FRM would have an unacceptable impact on the real estate value of affected landowners.

### MDR model

The MDR ologit model was significant (*p* = 0.0005) and explained a reasonable amount of variance for social models according to the McKelvey and Zavoinas R^2^ of 0.22 (Table 3). Six of 14 variables were significant if setting a threshold at *p* < 0.1. Four psychological variables were significant predictors of resistance to managed dyke realignment if homes or businesses needed to be relocated. The other-orientation statement that everyone should be treated equally was positively associated (0.281, *p* < 0.05), as (more weakly) was the past-orientation statement about having a feeling of having missed out on the good things in life (0.382, *p* < 0.1). Futures consciousness was negatively associated (meaning less resistance) (-0.796, *p* < 0.05), as was an awareness of species becoming extinct (-0.708, *p* = 0.001). Having heard of MDR was weakly negatively associated with resistance (-0.654, *p* < 0.1), as was being a male (-0.831, *p* < 0.05).

### FRM model

The FRM ologit model was significant but slightly weaker than the MDR one (*p* = 0.0016) and required more variables to achieve it. The FRM model also explained a reasonable amount of variance according to the McKelvey and Zavoinas R^2^ of 0.225 (Table 3). Only four of the 19 variables were significant. Two psychological variables were significant: the self-orientation statement about environmental action “threatening jobs for people like me” was weakly positively associated (0.397, *p* < 0.1), and futures consciousness was negatively associated (-0.753, *p* < 0.05). A belief that a flood in the next five years would cause damage to their home was positively associated with resistance (0.641, *p* < 0.05), as was having grandchildren (0.798, *p* < 0.05).

The significant grandchildren variable result was opposite to our expectations, so we did additional analyses to try and understand it further. Having grandchildren was not significantly associated with increased belief that flood risk would harm self or loved ones (Mann-Whitney) or increased free-market preferences (t-test). But there were some demographic variables (some of which had too much missingness to use in our full models) that suggest having grandchildren is partially associated with higher age (62.9 compared with 48.5 for those without grandchildren, Welch t-test *p* < 0.000), lower incomes (Mann-Whitney *p* = 0.008), and lower education (Mann-Whitney *p* = 0.0079). Yet when we ran a pairwise regression only with respondents who gave age and income on the flood model, while model power remained low due to the smaller n, the grandchildren variable was still significant. As a result, our findings suggest that the grandchildren variable is capturing something not otherwise explained by the variables we have.

## Discussion

We set out to explore resistance to two very different coastal climate adaptations in the Minas Basin of the Bay of Fundy using variables operationalizing climax thinking, along with issue awareness variables and demographic controls. The contrasting results, except for the future consciousness variable, speak to the importance of exploring adaptation options individually.

### Support for coastal climate adaptations

Both MDR and publicly available FRM were generally supported by those surveyed, although prior to the survey respondents were not well informed about either. Given this lack of awareness, participants unsurprisingly gave positive responses to statements describing potential positive impacts, even when they might seem to contradict. For instance, most respondents agreed that dykes should be raised to counter storm surge and sea level rise, *and* that restoring tidal wetlands would help protect Nova Scotia’s coastlines. Support for both options may not be as contradictory as they first appear but rather demonstrate that respondents see that the system may require a mix of protections, depending on the specific site. Moreover, such answers may bode well for ideas that combining stronger dykes and tidal wetland restoration, as done in MDR, is a robust option. Our findings are consistent with findings from Sutton (2020), who found that Nova Scotians in focus groups were reluctant to choose a preferred nature-based coastal adaptation as they felt each site was unique and required a unique adaptation. The vast majority of respondents agreed that publicly available FRM provides important information for current and future home buyers and renters, consistent with research from Raikes et al. (2023), who found that policy instruments targeted at communicating and reducing flood risk have high acceptability among Canadians, although property values are a significant concern (Oubennaceur et al. 2022).

Despite broad support, a minority of respondents were concerned by strategies if they involved relocation (20%, MDR) or loss of real estate value (30%, FRM). The resistance to FRM found in this study was twice as high as in previous work in another region of Nova Scotia that also asked about real estate value (Howard and Sherren 2023). There is mixed evidence that public FRM decreases property values (Hino and Burke 2021). For instance, researchers in Australia and Korea found that the release of public flood risk maps showing potential flood risk had minimal effect on property values compared with being in a flooded area (Rajapaksa et al. 2016; Seo et al. 2020). Mallette et al. (2021) found that across the literature, there is a public desire for governments to retain the current shoreline to protect private property. Assessments of the impact of mapping on real estate value have not yet been undertaken in this region because public flood-risk mapping is at such an early stage, although a range of Federal and Provincial mapping initiatives are underway.

Concern for loss of value due to potential impacts on homes or businesses is a well-documented finding in studies exploring public support for coastal resiliency projects. In order for these to succeed, most researchers find that there needs to be compensation for and recognition of the losses for impacted landowners (de la Vega-Leinert et al. 2018; Jones et al. 2014; Ledoux et al. 2005; Rulleau et al. 2017). In Nova Scotia, MDR projects typically include landowner consent and market-value compensation, but this may not be widely known, and it was not included in the brief description of MDR that respondents viewed before answering the questions. Further work could present scenarios, perhaps using vignette approaches (Sherren et al. 2021a), to explore conditions under which such relocations would be publicly acceptable.

### Drivers of resistance to coastal climate adaptations

The ologit models both had reasonable explanatory power for social models, especially given the complexity of the issues. Given space on a more targeted survey, additional variables could be added to fully explain resistance to these and other coastal climate adaptations. The work of scholars such as van Valkengoed and Steg (2019) and Bax et al. (2025) suggests a range of other variables that could be added in future work. For instance, for both MDR and FRM, public trust in governments and decision makers was found by other researchers to be an important influence in the acceptability and success of these projects around the world (Myatt et al. 2003; Roca and Villares 2012). However, the parallel models do give us some insight on additional variables researchers might consider, inspired by climax thinking, and they also demonstrate how each adaptation is likely to have different drivers depending on its impacts and proximity relative to personal circumstances. It is also worth noting that variables that would likely be significant in more politically polarized jurisdictions, such as climate change concern and free-market preferences, were insignificant in both our models.

Only one variable was significant in both models: being future-oriented was associated with lower resistance. It makes sense that a lack of future-orientation would be problematic in the face of climate adaptations like these. Previous research has shown that people who are future focused are generally more open and adaptable to change, and have less connection to past and historical landscape features (von Haaren 2002). Research in the Netherlands and the UK by Većkalov et al. (2021) found that those who are able to perceive future impacts of climate change are more inclined to accept long-term climate solutions that may not have immediate benefit. This is consistent with climax thinking (Sherren 2021), but does not illuminate whether the cause is exceptionalism or uncertainty. That is, we don’t know if the root cause is the discounting of future generations or a lack of understanding of how current actions affect them. While changing personalities to be more future-oriented is difficult, it *is* possible to inspire people to think of the future to reduce resistance during the planning and implementation of coastal climate adaptations. In a Delphi study of US climate adaptation workshop facilitators, more favored framing communication around desired future conditions than around immediate impacts, although most saw the choice as context-dependent (Stern et al. 2020). Sherren et al. (2022) also found that future priming was effective in encouraging openness to nature-based coastal climate adaptations in Nova Scotia, as prompted by reflection about duties to future generations. Combined with our results, it seems clear that a future-focused communicative priming strategy could be an important tool in reducing resistance to coastal climate adaptations.

### Public flood-risk mapping

The importance of a future-orientation on support for climate adaptation runs somewhat counter to another of the FRM model results: that those with grandchildren are *more* resistant to public flood-risk maps than those without. A pilot study in a nearby region had asked whether respondents had children, but that variable was so insignificant as to be removed from the final models (Howard and Sherren 2023). We asked about both children and grandchildren on this survey, but the former was again insignificant so was discarded early on in modelling. We maintained the grandchildren variable, expecting wrongly that having grandchildren would reduce resistance. Recently, Voorintholt et al. (2025) found similarly to us, using UK panel data, that there is no support for the idea that grandparents have higher climate change concern for the benefit of grandchildren. It remains a surprise that they would have higher resistance to public flood-risk mapping, given the barriers to home ownership among the young people they may care about. We assume that the variable is acting as a proxy for other characteristics that we did not capture on the survey. Age, income and education were not significant predictors, but it may still be that we are seeing the fear associated with losing home value—which is for many their biggest investment—*after* they have finished with salaried work. They may be relying on profits from the sale of their property to support them through their final years.

The final significant variables in the FRM model suggest resistance among those who think it is likely that a flood that occurs in the next five years would cause significant damage to their home, and/or those who agree that “protecting the environment will threaten jobs for people like me” (one of our self-orientation measures). The first suggests that those who perceive their property to be most at risk of flooding may not want their risk status to be publicly known, due to fear about the impacts on real estate values. A similar flood risk perception variable was not significant in a previous study of resistance to public FRM (Howard and Sherren 2023), but this risk perception paradox is well described by others. For instance, Wachinger et al. (2013) found in a review of natural disaster preparedness that those with high risk perception often still choose not to act. The second is consistent with Howard and Sherren (2023) who found using different measures that being self-concerned was one of the most significant drivers of resistance to publicly available FRM because of risks to real estate value. The significant measure used on this survey was related to environmental action negatively affecting “jobs for people like me”, which may have triggered similar concerns around income. Together the FRM model tells quite a coherent story about financial worries as a barrier to public interest information such as FRM, meaning such initiatives may be as contested in economically marginal regions like rural Nova Scotia as in places with higher-value real estate. A strong future focus may be the only remedy for the public and political resistance that emerges.

### Managed dyke realignment

Resistance to MDR has different predictors than resistance to FRM, save the lack of future-orientation already discussed. Looking first at the non-climax thinking variables, those who had heard previously of MDR, and/or self-described as male, were less resistant. These insights agree with those from an overlapping area by Sherren et al. (2016), which used Q-method to explore discourses around managed dyke realignment. A strong minority discourse in that study was a male-dominated one in support of dyke realignment and wetland restoration for reasons of system efficiency, in contrast with the dominant female discourse of dyke reinforcement for (agri)cultural purposes. If such individuals are already aware of MDR, they will also have less fear of the unknown than those for whom the idea is new. Such novelty is well known to drive resistance in other landscape change contexts such as renewable energy development (Enserink et al. 2022). As MDR becomes more common, resistance should be reduced through familiarity (Sherren et al. 2019).

The remaining significant variables predicting resistance to MDR tell interesting stories. For instance, resistance is higher among those who feel that they missed out on good things in their life, but not among those who feel they have happy childhood memories (this was originally included but removed from the MDR model during refinement but was not significant). This means that resistance to change because of a focus on the past might occur as much because that past was negative as because it was positive. Consistent with this sense of loss as a driver of climax thinking, Sherren et al. (2022) found that using a past-based priming in experimental focus groups did not reduce (and may have exacerbated) resistance to nature-based adaptation. The past priming seemed to remind people of what they had already lost, making them loath to lose more through landscape change. This reinforces the need to keep people focused on the future rather than the past when climate adaptation is being discussed.

Nature-orientation (specifically an awareness of species loss) was a significant negative predictor for opposition to MDR likely because MDR typically results in wetland restoration. While a nature dimension was not originally included in the climax thinking framework, it can be considered as part of the ‘other’ dimension, especially given more-than-human discourses emerging in wetland settings (Hurst et al. 2025). Tidal wetland cover around the Nova Scotia coast has been on a downward trajectory since Acadian settlement (Sherren et al. 2021b), prompting the development of a no-net-loss Provincial policy and related wetland compensation instruments. Those aware of species extinctions may feel that relocating homes and businesses is a fair trade-off for the benefits that will come from restoring such ecosystems (Sherren et al. 2025).

Finally, other-orientation was associated with MDR resistance, in contrast with the climax framework. But scale matters in interpreting this. The climax framework considered other-orientation as general ‘public good’ interests, such as MDR to modernize the dykeland system for all. An interest in solidarity with public interests has been shown by others to also be an important force in collective climate action, for instance to rebuild after flooding in Indonesia (Rahayu et al. 2022). But what we see in this model is that respondents are likely thinking about very specific people, people like them who may face relocation. This neighbourly concern may reflect the strong sense of community and history within the Bay of Fundy region, and particularly around its dykeland context (Eger and Courtenay 2021; Sherren et al. 2021b). As mentioned earlier, respondents to the survey are likely not aware of the fact that dyke realignments typically involve the consent of landowners impacted, and market-value land purchases (Sherren et al. 2019).

### Implications for climax thinking

Climax thinking is a novel framework for exploring the sources of resistance to coastal climate adaptation. Some of the variables that emerge as significant in predicting resistance here are consistent with conceptualizations of climax thinking as a barrier to climate action (Sherren 2021), especially the lack of future-orientation. Such drivers are also consistent with emerging frameworks such as futures consciousness, which we drew on in this work (Ahvenharju et al. 2018). But what we are also learning by this comparison is that one likely should deploy climax variables only when seeking to understand resistance to local landscape changes that are more immediate and visible. Upon reflection, neither of the adaptations fit that bill perfectly. We expected that dykeland changes would feel proximate to many in the region given their local salience (Sherren et al. 2025), but it may be only if a respondent lives near dykelands that they are likely to feel that MDR presents a change to their everyday landscape. Flood risk mapping will not directly change the appearance of the landscape but rather the sense of opportunities and risks it presents. A recent study in Belgium found that climax thinking (which they operationalized as a focus on the present rather than the past or future) was not predictive of support or opposition to blue economy developments, but similarly the proximity, impact and novelty of the presented options were not considered (Schmid et al. 2026). We thus need research on variables such as proximity, landscape impact and novelty to advance climax thinking. Relatedly, it may also be important to consider construal-level theory (Trope and Liberman 2010), which describes how psychological distance (as well as physical) can lead to more abstract thinking, whereby an adaptation can be supported in general but not near home. People may support managed dyke realignment while it remains an abstract concept, but their opinion may change once it reaches the planning or implementation stage in a specific location to which they feel attached.

Measures of climax thinking can also be improved. Parallel research suggests that a better approach to capturing climax thinking is directly asking about landscape perceptions. Margeson (2026) found that responses to the statement, “My landscape is pretty much in its optimal state right now”, increase the strength of modelled support for (and opposition to) tidal river restoration from pseudo R^2^ of 0.288 to 0.441. This is clearly a promising direction for research on climax thinking.

An additional direction of enquiry for climax thinking, which we were unable to address here, might be to further explore the impact of ancestors. Having grandchildren was significant in the FRM model, suggesting there are relational dimensions present. Ancestor-related statements have been included in the novel climax thinking question sets used previously (e.g., “Mine is the kind of coastal landscape that previous residents were working towards”; Sherren et al. (2022) but have not provided explanatory value. Yet, Eaton et al. (2021) found using qualitative methods that some landholders in their Pennsylvania study, presented with opportunities to host renewable energy, “felt a moral obligation to continue managing their land in ways they believed prior generations of their family would expect them to” (p. 237). This neglected dimension of embodied landscape norms may require further attention, particularly in rural areas where generational land management might influence climate decision-making.

### Limitations

Some important limitations bear mentioning. The lack of representativeness of the survey respondents relative to the demographics of the Minas Basin region, including gender, limits our capacity to generalize to the broader population. Better representation of lower-income people might have helped us to understand if opposition was associated with a difficulty meeting current needs, as might be suggested by Maslow (Upreti 2023). Our challenges with representativeness may have been caused in part by the length of the survey. This was an omnibus survey designed to inform several projects in the region, so was both longer and more complex than ideal when trying to achieve the widest representation. Researchers following this exploratory work should have a shorter list of constructs to include. Our models also suffered from missing data (leaving 178 to 180 model observations out of 217 respondents) that could introduce bias if the omissions were systematic. This is a known limitation of complete-case analysis. Finally, we acknowledge some ambiguity in the wording of the statements used as dependent variables in our models. While we take heart in their normal distributions, these may have been interpreted inconsistently across respondents. Future researchers should take care with their phrasing to ensure construct validity.

## Conclusions

This exploratory study sought to understand the knowledge and acceptance of two important climate adaptation options in a vulnerable coastal area, managed dyke realignment and public flood risk mapping. Most respondents were unfamiliar with both. We deployed an emerging climax thinking framework to statistically explore the reasons for the minority of resistance that emerged where there was risk to homes or real estate value. The only climax dimension that was predictive in both ologit models was future-orientation (which reduced resistance), supporting the growing consensus about the importance of prompting futures thinking in the face of climate change. Other significant variables differed, painting very different pictures of resistance depending on the specific adaptation, such as how close it is felt to be and the impacts it is felt to have for themselves and those they care about. Real estate is a clear concern for both adaptations, so robust analyses of potential impacts on both should precede public engagement. Evidence from this work suggests that climax thinking may be best used at the hyper-local scale, when impacts are close, visible and possibly novel. Future researchers should try to achieve more representative samples, given the sense that vulnerability is an important dimension, and experiment with different measures and variables in a wider range of settings and adaptations.

## Supplementary Information

Supplementary Material 1.

## Data Availability

The quantitative dataset for this study is publicly available in the open repository Borealis: https://doi.org/10.5683/SP3/G9USZ6.

## References

[CR1] Adger W, Arnell N, Tompkins E (2005) Successful adaptation to climate change across scales. Glob Environ Change 15(2):77. 10.1016/j.gloenvcha.2004.12.005

[CR2] Ahvenharju S, Minkkinen M, Lalot F (2018) The five dimensions of Futures Consciousness. Futures 104:1–13. 10.1016/j.futures.2018.06.010

[CR4] Bax V, van de Lageweg WI, Terpstra T, Buijs J-M, de Reus K, de Groot F, van Schaik R, Habte MA, Schram J, Hoogenboom T (2023) The impact of coastal realignment on the availability of ecosystem services: gains, losses and trade-offs from a local community perspective. J Environ Manage 345:118675. 10.1016/j.jenvman.2023.118675 37517096

[CR3] Bax V, Terpstra T, van de Lageweg WI, Buijs J-M, Filatova T (2025) Emotions, trust, and expectations: comparing determinants of public support for managed realignment across cases. J Environ Manage 380:125140. 10.1016/j.jenvman.2025.125140 40174392

[CR5] Bleakney JS (2004) Sods, soil, and spades the Acadians at Grand Pré and their dykeland legacy. McGill-Queen’s University. 10.1515/9780773572393

[CR6] Bush E, Lemmen DS (2019) Canada, & Environment and Climate Change Canada. Canada’s changing climate report. http://publications.gc.ca/collections/collection_2019/eccc/En4-368-2019-eng.pdf

[CR7] Chappell EN, Parkins JR, Sherren K (2020) Climax thinking, place attachment, and utilitarian landscapes: implications for wind energy development. Landsc Urban Plann 199:103802. 10.1016/j.landurbplan.2020.103802

[CR8] datazONE (2022) Residential Dwelling Characteristics, DatazONE, Dataset. https://www.thedatazone.ca/Assessment/Residential-Dwelling-Characteristics/a859-xvcs

[CR9] de la Vega-Leinert AC, Stoll-Kleemann S, Wegener E (2018) Managed Realignment (MR) along the eastern German Baltic Sea: a catalyst for conflict or for a coastal zone management consensus. J Coastal Res 34(3):586. 10.2112/JCOASTRES-D-15-00217.1

[CR10] Dillman D (1978) Mail and telephone surveys: The total design method. Wiley

[CR11] Eaton WM, Hinrichs CC, Burnham M (2021) Cultural counterpoints for making sense of changing agricultural and energy landscapes. Energy Impacts: a multidisciplinary exploration of north American energy development. University Press of Colorado, p 215

[CR12] Eger SL, Courtenay SC (2021) Integrated coastal and marine management: insights from lived experiences in the Bay of Fundy, Atlantic Canada. Ocean Coastal Management 204:105457. 10.1016/j.ocecoaman.2020.105457

[CR13] Enserink M, Van Etteger R, Van Den Brink A, Stremke S (2022) To support or oppose renewable energy projects? A systematic literature review on the factors influencing landscape design and social acceptance. Energy Research Social Science 91:102740. 10.1016/j.erss.2022.102740

[CR14] Gärling T, Fujii S, Gärling A, Jakobsson C (2003) Moderating effects of social value orientation on determinants of proenvironmental behavior intention. J Environ Psychol 23(1):1–9. 10.1016/S0272-4944(02)00081-6

[CR15] Glavovic B, Dawson R, Garschagen M, Haasnoot M, Singh C, Thomas A (2022) Cities and settlements by the sea. In: Pörtner H, Roberts D, Tignor M, Poloczanska E, Mintenbeck K, Alegría A, Craig M, Langsdorf S, Löschke S, Möller V, Okem A, Rama B (eds) Climate Change 2022 – impacts, adaptation and vulnerability: working group II contribution to the sixth assessment report of the Intergovernmental Panel on Climate Change, 1st edn. Cambridge University Press. 10.1017/9781009325844

[CR16] Hansla A, Gamble A, Juliusson A, Gärling T (2008) The relationships between awareness of consequences, environmental concern, and value orientations. J Environ Psychol 28(1):1–9. 10.1016/j.jenvp.2007.08.004

[CR17] Harlan SL, Sarango MJ, Mack EA, Stephens TA (2019) A survey-based assessment of perceived flood risk in urban areas of the United States. Anthropocene 28:100217. 10.1016/j.ancene.2019.100217

[CR18] Henstra D, Minano A, Thistlethwaite J (2019) Communicating disaster risk? An evaluation of the availability and quality of flood maps. Nat Hazards Earth Syst Sci 19(1):313–323. 10.5194/nhess-19-313-2019

[CR19] Hino M, Burke M (2021) The effect of information about climate risk on property values. Proc Natl Acad Sci 118(17):e2003374118. 10.1073/pnas.2003374118 PMC8092410 33879604

[CR20] Hirabayashi Y, Mahendran R, Koirala S, Konoshima L, Yamazaki D, Watanabe S, Kim H, Kanae S (2013) Global flood risk under climate change. Nat Clim Change 3(9):816–821. 10.1038/nclimate1911

[CR21] Howard SC, Sherren K (2023) Flood risk mapping in southwestern Nova Scotia: perceptions and concerns. The Can Geographer / Le Géographe Canadien. 10.1111/cag.12836

[CR22] Hügel S, Davies AR (2020) Public participation, engagement, and climate change adaptation: a review of the research literature. WIREs Clim Change 11(4):e645. 10.1002/wcc.645 PMC9285715 35859618

[CR23] Hurst E, Ellis R, Karippal AB (2025) Lively water infrastructure: constructed wetlands in more-than-human waterscapes. Environ Plann E: Nat Space 8(1):77–99. 10.1177/25148486221113712

[CR24] International Union for Conservation of Nature (2020) IUCN global standard for nature-based solutions: 1st edn. IUCN. https://portals.iucn.org/library/node/49070

[CR25] IPCC (2023) Climate Change 2023: synthesis report, 1st edn. Intergovernmental Panel on Climate Change (IPCC). 10.59327/IPCC/AR6-9789291691647

[CR26] Jackson H (2019) National climate emergency declared by House of Commons. *Global News*. https://globalnews.ca/news/5401586/canada-national-climate-emergency/

[CR27] James TS, Henton JA, Leonard LJ, Darlington A, Forbes DL, Craymer M (2015) Tabulated values of relative sea-level projections in Canada and the adjacent mainland United States. Geological Survey of Canada, Open File, 7942, 81. Natural Resources Canada. 10.4095/297048

[CR28] Jones N, Koukoulas S, Clark JRA, Evangelinos KΙ, Dimitrakopoulos PG, Eftihidou MO, Koliou A, Mpalaska M, Papanikolaou S, Stathi G, Tsaliki P (2014) Social capital and citizen perceptions of coastal management for tackling climate change impacts in Greece. Reg Envriron Chang 14(3):1083–1093. 10.1007/s10113-013-0540-5

[CR29] Keller C, Bostrom A, Kuttschreuter M, Savadori L, Spence A, White M (2012) Bringing appraisal theory to environmental risk perception: a review of conceptual approaches of the past 40 years and suggestions for future research. J Risk Res 15(3):237–256. 10.1080/13669877.2011.634523

[CR30] Lalot F, Ahvenharju S, Minkkinen M, Wensing E (2020) Aware of the future? Development and validation of the futures consciousness scale. Eur J Psychol Assess 36(5):874–888. 10.1027/1015-5759/a000565

[CR31] Ledoux L, Cornell S, O’Riordan T, Harvey R, Banyard L (2005) Towards sustainable flood and coastal management: identifying drivers of, and obstacles to, managed realignment. Land Use Policy 22(2):129–144. 10.1016/j.landusepol.2004.03.001

[CR32] Lyle TS, Fang LL, Hund SV (2024) Implications of disclosure and non-disclosure of flood hazard maps – a synthesis for the Canadian context. Canadian Water Resour Journal / Revue Canadienne Des Ressources Hydriques 0(0):1–18. 10.1080/07011784.2023.2287462

[CR33] MacDonald M, Smellie S (2021) Atlantic storm: flooding in Cape Breton and N.L. CTV News. https://www.ctvnews.ca/canada/flooding-reported-in-cape-breton-and-western-newfoundland-as-storm-stalls-over-region-1.5678999

[CR34] Mallette A, Smith TF, Elrick-Barr C, Blythe J, Plummer R (2021) Understanding preferences for coastal climate change adaptation: a systematic. Literature Rev Sustainability 13(15). 10.3390/su13158594

[CR35] Margeson K (2026) Using comparative social impact assessment to understand resistance and support for causeway removal and tidal river restoration. Dissertation, Dalhousie University.

[CR36] Myatt LB, Scrimshaw MD, Lester JN (2003) Public perceptions and attitudes towards a forthcoming managed realignment scheme: Freiston Shore, Lincolnshire, UK. Ocean Coastal Management 46(6–7):565–582. 10.1016/S0964-5691(03)00035-8

[CR37] Myers T, Maibach E, Roser-Renouf C, Akerlof KL, Leiserowitz A (2013) The relationship between personal experience and belief in the reality of global warming. Nat Clim Change 3:343–347. 10.1038/NCLIMATE1754

[CR38] NOAA (2023) JetStream Max: Bay of Fundy: the highest tides in the world. https://www.noaa.gov/ocean/fundy-max

[CR39] Oubennaceur K, Chokmani K, Lessard F, Gauthier Y, Baltazar C, Toussaint J-P (2022) Understanding flood risk perception: a case study from Canada. Sustainability 14(5):3087. 10.3390/su14053087

[CR40] Rahayu I, Irwan I, Putra IM (2022) Supporting factors for the development of community social solidarity in the face of the flood disaster in Sungai Limau district, Padang Pariaman regency, West Sumatera. Jurnal Analisa Sosiologi 11(4). 10.20961/jas.v11i4.63832

[CR41] Raikes J, Henstra D, Thistlethwaite J (2023) Public attitudes toward policy instruments for flood risk management. Environ Manage. 10.1007/s00267-023-01848-3 PMC10509047 37392239

[CR42] Rajapaksa D, Wilson C, Managi S, Hoang V, Lee B (2016) Flood risk information, actual floods and property values: a quasi-experimental analysis. Economic Record 92(S1):52–67. 10.1111/1475-4932.12257

[CR43] Roca E, Villares M (2012) Public perceptions of managed realignment strategies: the case study of the Ebro Delta in the Mediterranean basin. Ocean Coastal Management 60:38–47. 10.1016/j.ocecoaman.2012.01.002

[CR44] Rudin R (2021) Against the tides: Reshaping landscape and community in Canada’s maritime marshlands. UBC. https://www.ubcpress.ca/against-the-tides

[CR45] Rulleau B, Rey-Valette H, Clément V (2017) Impact of justice and solidarity variables on the acceptability of managed realignment. Clim Policy 17(3):361–377. 10.1080/14693062.2015.1119097

[CR46] Sarabi S, Han Q, Romme AGL, de Vries B, Valkenburg R, den Ouden E (2020) Uptake and implementation of Nature-Based Solutions: An analysis of barriers using Interpretive Structural Modeling. J Environ Manage 270:110749. 10.1016/j.jenvman.2020.110749 32721286

[CR47] Schmid M, Cauberghe V, Bouman T, Vandamme S (2026) Perceptions of the Belgian coast through time: how resident views affect acceptance of blue projects and place attachment. J Place Manage Dev 19(1):108–134

[CR48] Schmunk R (2021) A timeline of how once-in-a-century flooding unfolded across B.C. CBC. https://newsinteractives.cbc.ca/longform/bc-flooding-2021-timeline-how-once-in-a-century-flooding-unfolded

[CR49] Schwartz SH (1994) Are there universal aspects in the structure and contents of human values? J Soc Issues 50(4):19–45. 10.1111/j.1540-4560.1994.tb01196.x

[CR50] Schwartz SH (2012) An overview of the Schwartz Theory of Basic Values. Online Readings Psychol Cult 2(1). 10.9707/2307-0919.1116

[CR51] Seo J, Oh J, Kim J (2020) Flood risk awareness and property values: evidences from Seoul, South Korea. Int J Urban Sci 25(2):233–251. 10.1080/12265934.2020.1850324

[CR52] Sherren K (2021) From climax thinking toward a non-equilibrium approach to public good landscape change. In: Jacquet J, Haggerty J, Theodori G (eds) Energy impacts: a multidisciplinary exploration of North American energy development, Social Ecology Press & Utah State University, pp 17–44. 10.5876/9781646420278.c001

[CR57] Sherren K, Loik L, Debner J (2016) Climate adaptation in new world cultural landscapes: the case of Bay of Fundy agricultural dykelands (Nova Scotia, Canada). Land Use Policy 51:267–280. 10.1016/j.landusepol.2015.11.018

[CR53] Sherren K, Bowron T, Graham JN, Rahman HT, van Proosdij D (2019) Coastal infrastructure realignment and salt marsh restoration in Nova Scotia, Canada. In responding to rising seas: OECD country approaches to tackling coastal risks. OECD pp 111–135. 10.1787/dc713684-en

[CR54] Sherren K, Chappell E, Parkins J (2021a) Strategies for integrating quantitative methods into critical social acceptance research. In: Batel S, Rudolph D (eds) A critical approach to the social acceptance of renewable energy infrastructures. Springer International Publishing, pp 23–42. 10.1007/978-3-030-73699-6_2

[CR56] Sherren K, Ellis K, Guimond JA, Kurylyk B, LeRoux N, Lundholm J, Mallory ML, van Proosdij D, Walker AK, Bowron TM, Brazner J, Kellman L, Turner II, B. L., Wells E (2021b) Understanding multifunctional Bay of Fundy dykelands and tidal wetlands using ecosystem services—A baseline. FACETS 6:1446–1473. 10.1139/facets-2020-0073

[CR59] Sherren K, Sutton K, Chappell E (2022) Climax thinking on the coast: a focus group priming experiment with coastal property owners about climate adaptation. Environ Manage 70(3):475–488. 10.1007/s00267-022-01676-x PMC9381476 35773431

[CR58] Sherren K, Rahman HMT, Manuel P, Wells E, Rapaport E, Van Proosdij D (2024) Reimagining nature-based coastal adaptation: a nested framework. J Flood Risk Manag 17(4):e13026. 10.1111/jfr3.13026

[CR55] Sherren K, Cornejo L, McWherter B, Howard S, Legault A (2025) Proximity, benefit transfer and trade-offs: the limits of ecosystem service assumptions in an anthropogenic rural coastal setting. Ecosyst People 21. 10.1080/26395916.2025.2547597

[CR60] Starratt K (2022) Halls Harbour flooding illustrates dangers of sea level rise on Bay of Fundy communities. SaltWire. https://www.saltwire.com/atlantic-canada/news/halls-harbour-flooding-illustrates-dangers-of-sea-level-rise-on-bay-of-fundy-communities-100703284/

[CR62] Stern PC, Dietz T, Abel T, Guagnano GA, Kalof L (1999) A Value-Belief-Norm theory of support for social movements: the case of environmentalism. Hum Ecol Rev 6(2):81–97

[CR61] Stern MJ, Brousseau J, O’Brien C, Hurst K, Hansen LJ (2020) Climate adaptation workshop Delphi study report: facilitators’ viewpoints on best practices. Virginia Tech and EcoAdapt

[CR63] Sutton K (2020) Understanding perceptions of coastal climate change and nature-based coastal adaptation: Using communicative framing in experimental focus groups in Nova Scotia, Canada. https://DalSpace.library.dal.ca//handle/10222/80147

[CR64] Thistlethwaite J, Henstra D, Brown C, Scott D (2018) How flood experience and risk perception influences protective actions and behaviours among Canadian homeowners. Environ Manage 61(2):197–208. 10.1007/s00267-017-0969-2 29234832

[CR65] Trope Y, Liberman N (2010) Construal-level theory of psychological distance. Psychol Rev 117(2):440–463. 10.1037/a0018963 PMC3152826 20438233

[CR66] Upreti G (2023) Satisfaction of human needs and environmental sustainability. In: Upreti G (ed) Ecosociocentrism: the earth first paradigm for sustainable living. Springer Nature Switzerland, pp 111–135. 10.1007/978-3-031-41754-2_6

[CR67] van der Meulen F, IJff S, van Zetten R (2022) Nature-based solutions for coastal adaptation management, concepts and scope, an overview. Nord J Bot 2023. 10.1111/njb.03290

[CR68] van Proosdij D, Ross C, Matheson G (2018) Nova Scotia Dyke Vulnerability Assessment. Nova Scotia Department of Agriculture

[CR69] van Valkengoed AM, Steg L (2019) Meta-analyses of factors motivating climate change adaptation behaviour. Nat Clim Change 9(2):158–163. 10.1038/s41558-018-0371-y

[CR70] Većkalov B, Zarzeczna N, Niehoff E, McPhetres J, Rutjens BT (2021) A matter of time… consideration of future consequences and temporal distance contribute to the ideology gap in climate change scepticism. J Environ Psychol 78:101703. 10.1016/j.jenvp.2021.101703

[CR71] von Haaren C (2002) Landscape planning facing the challenge of the development of cultural landscapes. Landsc Urban Plann 60(2):73–80. 10.1016/S0169-2046(02)00060-9

[CR72] Voorintholt L, Van Den Berg GJ, Soetevent AR (2025) For the future of our grandchildren: grandparenthood and climate change concerns. SSRN Electron J. 10.2139/ssrn.5194042

[CR73] Vouk I, Pilechi V, Provan M, Murphy E (2021) Nature-based solutions for coastal and riverine flood and erosion risk management. p 69

[CR74] Wachinger G, Renn O, Begg C, Kuhlicke C (2013) The risk perception paradox—implications for governance and communication of natural hazards. Risk Anal 33(6):1049–1065. 10.1111/j.1539-6924.2012.01942.x 23278120

[CR75] Wamsler C (2018) Mind the gap: the role of mindfulness in adapting to increasing risk and climate change. Sustain Sci 13. 10.1007/s11625-017-0524-3 PMC6086299 30147799

[CR76] Esteves LS, Williams JJ (2017) Managed realignment in Europe: a synthesis of methods, achievements and challenges. In: Bilkovic DM, Mitchell MM, Toft JD, La Peyre MK (eds) Living Shorelines: the science and management of nature-based coastal protection. CRC Press, Taylor & Francis Group, p 157-180. 10.1201/9781315151465-11

[CR77] Zimbardo PG, Boyd JN (1999) Putting time in perspective: a valid, reliable individual-differences metric. J Personal Soc Psychol 77(6):1271–1288. 10.1037/0022-3514.77.6.1271

