# Supplementary material for: Using climax thinking to understand psychological drivers of attitudes towards two coastal climate adaptations around the hypertidal Bay of Fundy
Source: Anthr Coasts. 2026 Oct 30;9(1):46. doi: 10.1007/s44218-026-00163-8 (PMC13739002; doi:10.1007/s44218-026-00163-8)
Supplement: Supplementary file 1 [file 44218_2026_163_MOESM1_ESM.pdf]

doi: 10.1007/s44218-026-00163-8

Supplemental Table 1: Demographics of surveyed sample compared to demographics of the 2021 Canadian Census for the counties of Hants, Kings, Cumberland, and Colchester.

**Topic**	**Category**	**Census (% of total)**	**Survey (% of total)**
Income	Under $10,000	8%	0
	$10,000 to $19,999	14%	1%
	$20,000 to $29,999	18%	5%
	$30,000 to $39,999	14%	9%
	$40,000 to $49,999	13%	7%
	$50,000 to $59,999	9%	8%
	$60,000 to $69,999	7%	13%
	$70,000 to $79,999	5%	11%
	$80,000 to $89,999	4%	6%
	$90,000 to $99,999	3%	8%
	$100,000 to $149,999	4%	17%
	$150,000 and over	1%	15%
Age	20 to 24	6%	2%
	25 to 29	6%	6%
	30 to 34	7%	2%
	35 to 39	7%	7%
	40 to 44	7%	11%
	45 to 49	8%	11%
	50 to 54	9%	6%
	55 to 59	10%	11%
	60 to 64	10%	21%
	65 to 69	9%	11%
	70 to 74	8%	5%
	75 to 79	6%	5%
	80+	7%	2%
Gender	Male	49%	34%
	Female	51%	66%
Education	High school or lower	41%	12%
	Some college	39%	28%
	Some university	20%	60%

doi: 10.1007/s44218-026-00163-8

a)

Dykeland is the land that was created out of tidal wetlands by building earthen walls to keep out tides, thus draining them of salt water. Dykeland was originally designed for farming but many different land uses exist in dykeland today.

Dykes are the linear earthen walls that protect dykeland. Dykes are interrupted every so often by aboiteaux that help to drain rainwater from dykeland during low tide to keep it dry.

Tidal wetland is not always in front of dykes as shown here, but comprises vegetated areas that are flooded to some degree by regular tides.

Managed dyke realignment is the process of building higher dykes further inland from current ones and restoring the former dykeland in front of them back to tidal wetlands. Below is a photograph of a recent managed realignment project at Belcher Street, along the Jijuktu'kewjk/Cornwallis River, near Kentville, NS, where a new dyke has been built to continue to protect active farmland. Some former dykeland areas in front of the new dyke are being restored to tidal wetland.

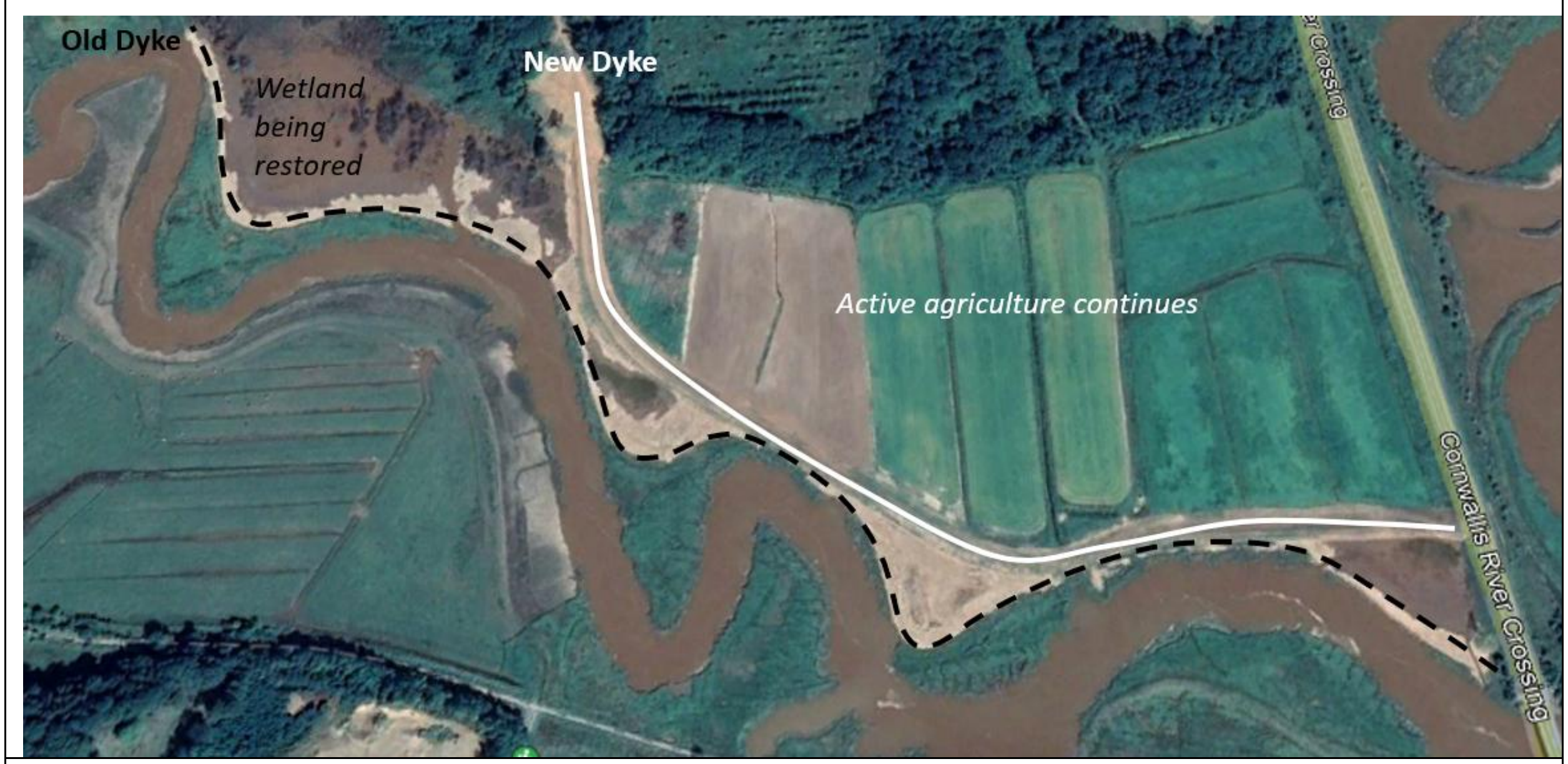

b)

A **flood risk map** is a map that shows an area of land, homes, roads, buildings, and other infrastructure that may be at risk in a specified region in the event of a flood. Below is an example of a flood risk map in the City of Toronto during a 1 in 100 year flood event, which is a flood that has a 1% probability of occurring in any given year.

(image not available)

Supplemental Box 1: Wording of the coastal adaptation questions, including graphics provided in advance of each question set shown in Table 1.

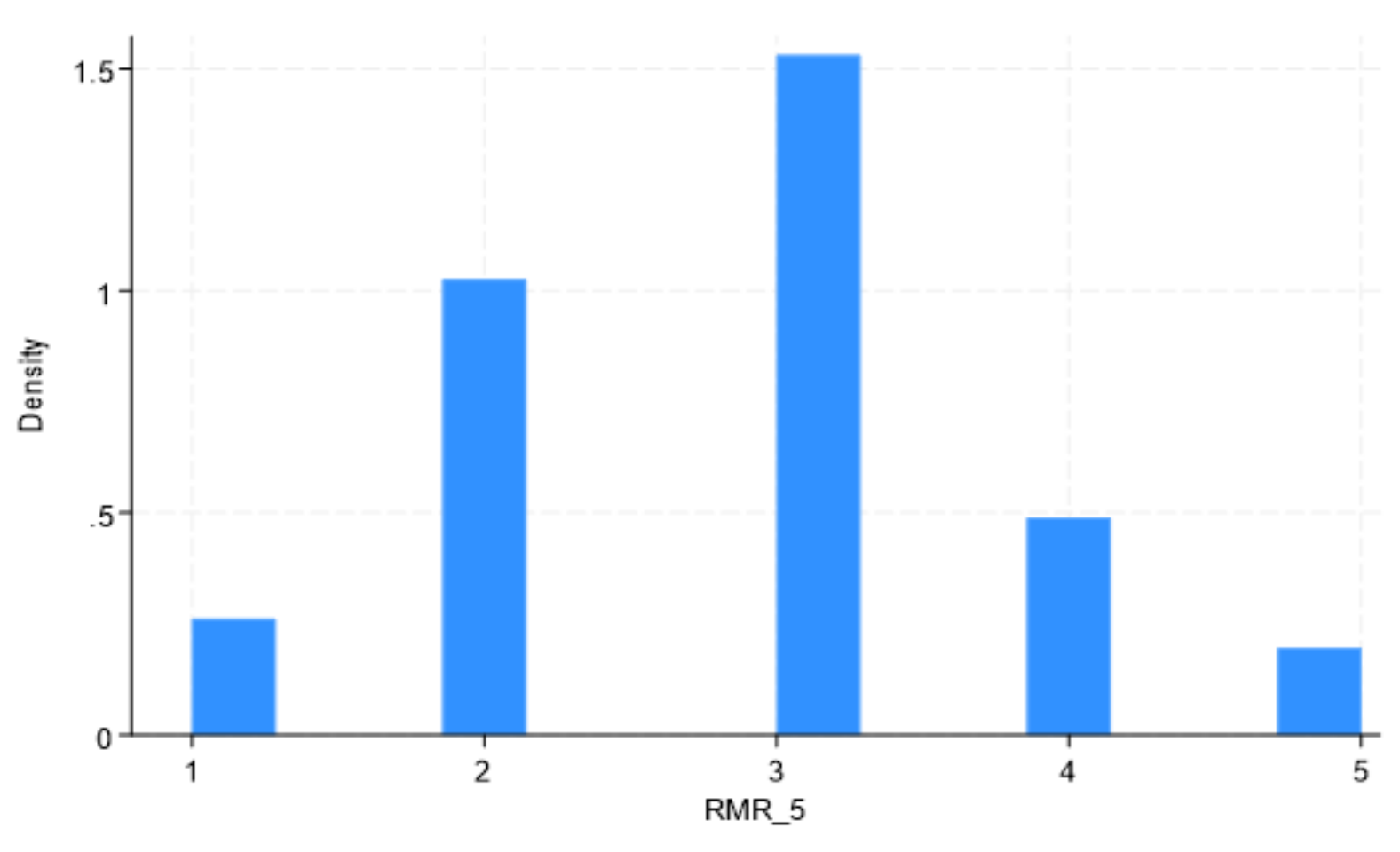

a) Resistance to managed realignment

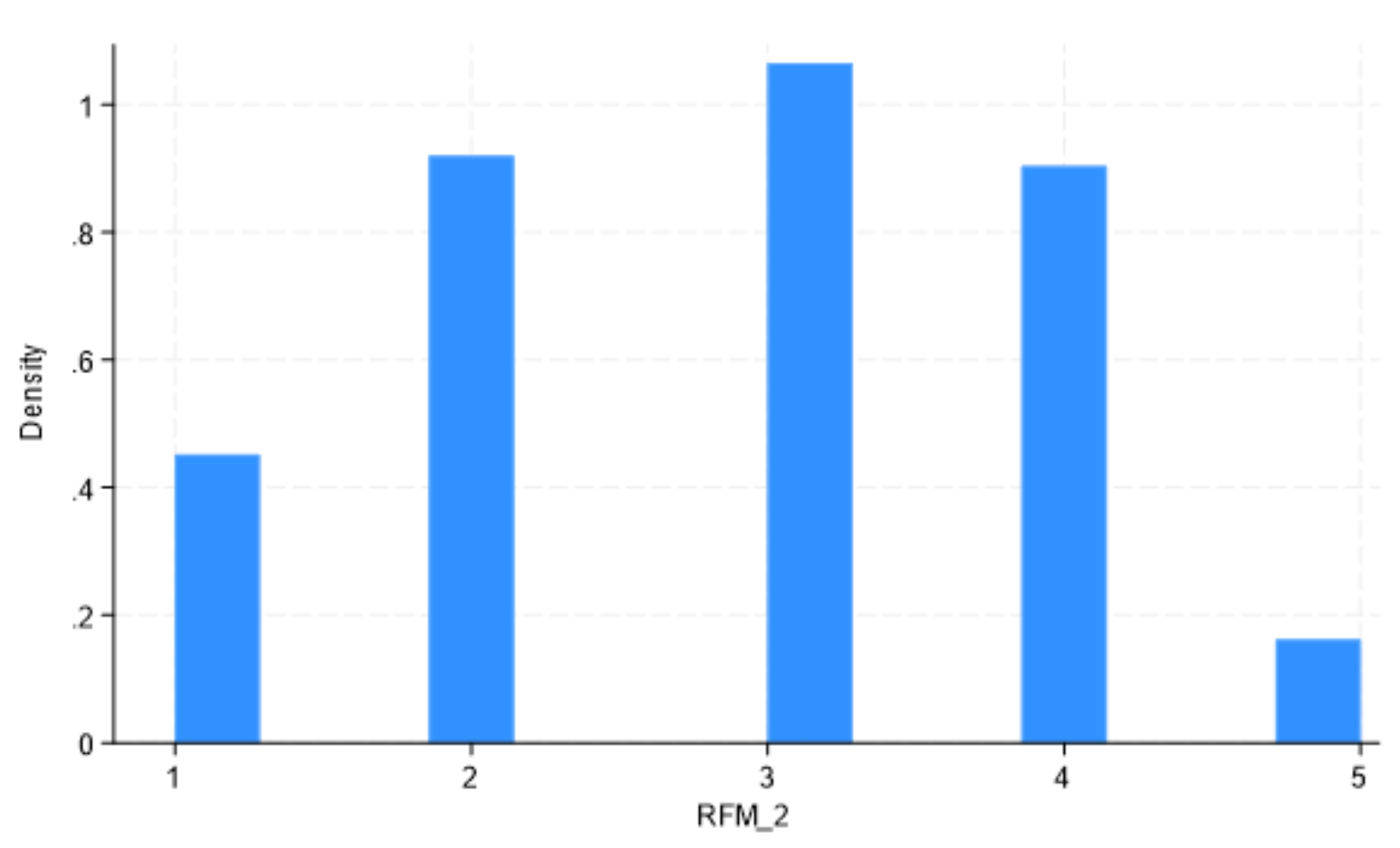

b) Resistance to public flood risk mapping.

Supplemental Figure1: Distribution of response variables (see Table 2)

doi: 10.1007/s44218-026-00163-8

Supplemental Table 2: Robustness checks for demographic variables to establish whether biases in the sample affected the response variable for a) MDR and b) FRM. Means are provided for continuous variables and proportions for categorical variables with F and Chi-Sq tests. GN=gender.

a)

	Managed Dyke Realignment					
	5	4	3	2	1	Test
N	16 (7.4%)	63 (29.3%)	94 (43.7%)	30 (14.0%)	12 (5.6%)	
AGE	53.667 (15.319)	52.704 (16.590)	52.049 (14.323)	57.533 (12.778)	55.833 (8.612)	0.775
INC	8.375 (2.655)	9.413 (2.993)	8.978 (3.448)	8.897 (3.166)	10.917 (2.065)	0.235
GN	1.562 (0.512)	1.556 (0.532)	1.793 (0.504)	1.733 (0.450)	1.583 (0.515)	0.043
EDU	4.875 (1.784)	4.746 (1.534)	4.946 (1.924)	5.333 (1.626)	3.833 (1.403)	0.146

b)

	Flood Risk Mapping					
	1	2	3	4	5	Test
N	28 (12.9%)	57 (26.3%)	66 (30.4%)	56 (25.8%)	10 (4.6%)	
AGE	59.214 (17.080)	51.344 (14.548)	49.615 (15.618)	57.095 (10.324)	67.000 (8.206)	0.053
INC	9.286 (3.053)	9.618 (3.112)	9.121 (3.251)	8.745 (3.210)	8.111 (3.408)	0.546
GN	1.571 (0.504)	1.754 (0.474)	1.682 (0.469)	1.691 (0.605)	1.667 (0.500)	0.660
EDU	4.964 (1.795)	5.000 (1.524)	4.621 (1.936)	5.091 (1.636)	3.889 (2.315)	0.254

doi: 10.1007/s44218-026-00163-8

Supplemental Table 3 Descriptive statistics for the climate concern and flood risk perception variables.

**Statement and response options**	**n**	**Prop.**
How important is the issue of climate change to you personally?	215	
Extremely important		.39
Very important		.33
Somewhat important		.20
Not too important		.05
Not at all important		.02
How worried are you about climate change?	217	
Very worried		.45
Somewhat worried		.45
Not very worried		.07
Not worried at all		.03
How much do you think climate change will harm you personally?	206	
A great deal		.18
A moderate amount		.58
Only a little		.18
Not at all		.06
How much do you think climate change will harm future generations?	210	
A great deal		.80
A moderate amount		.15
Only a little		.02
Not at all		.02
We don't need to worry much about the environment because future generations will be better able to deal with these problems than we are	216	
Strongly agree		.01
Agree		.02
Neither agree nor disagree		.08
Disagree		.34
Strongly disagree		.55
Suppose a flood occurred in your region within the next five years. How likely do you think it would be that your home would be seriously damaged or destroyed?	213	
Extremely likely		.15
Somewhat likely		.46
Unlikely		.25
Not likely at all		.14
How likely do you think it would be that you or some of your loved ones would be hurt?	213	
Extremely likely		.10
Somewhat likely		.46
Unlikely		.36
Not likely at all		.09

doi: 10.1007/s44218-026-00163-8

Supplemental Table 4 Descriptive statistics for key psychological variables. See Table 1 for full wording and response options.

**Statement**	Dimension	n	Mean	SD	Min.	Max.
CC will threaten jobs	Self	216	1.87	.93	1.00	5.00
Laws to protect env limit personal freedom	Self	217	2.28	1.15	1.00	5.00
It is important everyone treated equally	Other	214	4.42	1.45	1.00	6.00
It is important to help others	Other	214	4.22	1.33	1.00	6.00
Think about future	Future	215	4.13	.65	1.00	5.00
Willing to sacrifice now to help future	Future	216	3.68	.83	1.00	5.00
Day to day to influence future	Future	215	3.99	.69	2.00	5.00
Think about good things missed in life	Past	216	2.59	.96	1.00	5.00
Familiar childhood sights create memories	Past	216	4.07	.84	1.00	5.00
Believe people should care for nature	Nature	213	4.44	1.31	2.00	6.00
Species go extinct	Nature	217	4.12	.84	1.00	5.00
